# Foreign Correspondence

**Published:** 1867-09

**Authors:** 


### Foreign Correspondence.

HEIDELBERG, July 14th, 1867.

DEAR DOCTOR:—For the present I find myself domiciled in this ancient and beautiful city of the valley of the Neckar. There is just room enough for the city between the river and mountain, which rises abruptly to the height of 1800 feet. On the opposite right bank is another range of similar height, springing from the very water's edge. At the lower limits of the city this narrow valley opens out on a plain, extending beyond the range of vision. This plain is under the highest state of cultivation, and owing to the tortuous course of the river is very accessible to its benefits. This assemblage of mountains, valley, and river, can be seen in "bird's-eye views" from any of the neighboring peaks, and is altogether the most charming natural *picture* that it has ever been my good fortune to witness elsewhere. Heidelberg has a population of 16,000 *humans*, and because of its being a popular resort for pleasure and sight-seeing, one finds almost every nation and tongue represented, particularly in public places. Its claims to antiquity crop out here and there in the architecture of olden times, represented by a few buildings that were spared by the incendiaries under Gen. Medoc, who led the French against it in 1693—Louis XIV. time. Among these reminiscences that were spared was a portion of the feudal castle, situated on the mountain side just above the city. It contains many curious relics of the past, such as old pictures, mostly representing Saints, or "God's anointed" tyrants, implements of warfare, and last, though not least, the famous old wine cask, 30 feet long and 28 feet in diameter, and capable of containing 300,000 bottles of wine. I did not take much interest in that "barrel," because it is quite empty now, and the bung hole does not even smell of the ghosts of good drinks that it once afforded.

The University, founded A.D. 1386, under the Elector Rupert III., is one of the most celebrated in Europe, consequently is the resort of students from all parts of the world. The usual number in attendance is about 800. They are for the most part jolly, rollicking looking fellows. I learned that their principal recreation is fighting duels with swords, and, owing to this, one may pick them out on the streets by the variegated scars on their faces. The above remark is only true of the seniors, the juniors (or "fuchses or foxes") have to bide their time for this badge of honor. One would look upon this custom as rather risky, until informed that every part of the body is protected by a coat of mail, excepting the cheeks. The eyes are covered by a pair of huge iron spectacles. A Sioux Indian is not prouder of a white man's scalp than one of these young rascals of his numerous cheek gashes—the fresher the better.

From force of habit, or because I understand it best, the medical department has most charms for me, in which I had the extreme pleasure of meeting my former pupil and friend, Dr. Samuel Cole, of Chicago, for whose kind attentions and valuable aid I am much indebted. Although young, the Doctor is already quite ripe in medical and surgical lore, having graduated at Rush Medical College, attended one year at Bellevue, one year in Paris, and already six months in this city. He informed me that he considers this institution much to be preferred to any other that he has visited, mainly because of the thorough method pursued in giving clinical instruction, which method, as I have learned by observation, is about as follows, viz.: The Clinical Professor selects in rotation each morning a different member of his class, who is directed to make the clinical examinations, embracing diagnosis, pathology, prognosis, etc., the teacher correcting all mistakes at the time. Of course this plan can only be pursued with a limited number of students, but, circumstances permitting, it is certainly very practical. The young men who enjoy these privileges are advanced students or graduates. Could not a similar plan be adopted in Rush Medical College for advanced stndents and young graduates, as an institution independent of the regular course of instruction, and made an appendage of the Dispensary? I send you a synopsis of a clinical lecture by the celebrated Prof. Friedrich, (Lec. on Theory and Practice,) as an example of the painstaking plan of examining cases for the instruction of student, and this is true of all the departments. In the above case, Prof. F. caused his selected pupil to repeat the anatomy of the entire nervous system at the base of the brain in arriving at an accurate diagnosis. Prof. Knapp, in his Eye Clinic, pursues the same severe analytical course, causing the pupil whose "turn it is," to hobble on before him, always holding himself in readiness to correct mistakes or to elucidate obscure points.

Owing to the poverty of the masses in this country there is always an abundance of clinical material, consequently their large hospital is always well filled with every variety of disease. I am aware that "comparisons are odious," but must, nevertheless, indulge somewhat respecting our surgery and theirs. For example, in the treatment of hip-joint disease, they make use of an immovable apparatus, consisting of a plaster of Paris case, which embraces both hips and the affected thigh, provided with convenient openings for the escape of excreta .and pus, in imitation of "Bauer's wire breeches." It is true this partially fulfills the indications of "mechanical and physiological iest," but the most important indication is unfulfilled, which is the separation of the joint surfaces by extension and counter extension, thereby counteracting the mischievous effects of the ever contracting muscles, at the same time (as in the use of Taylor's or Davis' elegant splints,) rendering it expedient for the patient to go about and receive the benefits of open air, moreover, offering the additional advantage of avoiding subsequent anchylosis. I had occasion to observe the same plan of treatment in caries of the knee and elbow, which must necessarily result in true anchylosis, if the cases happen to terminate so fortunately, in these merciless vices of plaster. The fact is, these Europeans could learn something regarding mechanical surgical treatment by coming to the United States, for in this department we are far in advance of them, while I am ready to acknowledge that in most purely scientific departments we must concede an inferiority, not that our endowments for scientific research are inferior, but time is too valuable with us to spend it in the comparative anatomy of the "germinal spot" of the egg of an itch-mite.

Their plastic operations excel in neatness of execution and success anything I have seen elsewhere, and one may add that their surgieal operations generally exhibit a great deal of skill. Weber, the surgeon, and a man of much eminence, died a few days since of diphtheria. His handiwork still adorns the hospital, in the form of plastic operations, resections, etc. Prof. Heine—a man of less celebrity, but perhaps of equal skill, fills his place until the regular appointment is made.

Heidelberg is the home of the celebrated surgeon, Chelius, (the author of Chelius' Surgery,) to whom I had the pleasure of an introduction. He is quite aged, being upwards of 80 years, and has retired from the profession, although still possessed of an active mind. He spoke very feelingly of Mott, for whom he seems to have entertained much respect. When I mentioned Brainard's death, he quickly signified that he was aware of it, and commenced conversing about his improvement in the treatment of ununited fractures, for which he gives him much credit.

Dear Doctor, I do not think I will be home before the last of October, as I have so much laid out to accomplish that it will consume all that time and more. I have not heard a word about the College since leaving home, but expect a letter from you hourly, in which I expect to find all the news I desire. Neither have I received the MEDICAL JOURNAL that you promised. Please send it along. I expect to go to Tübingen in a few days, in order to visit their medical school, when you will hear from me again, if I can gather anything of interest.

Respecting ourselves, we are all enjoying excellent health, and enjoy the city of our temporary adoption. My letter, you will notice, was commenced at Heidelberg, but it has been completed at home. I do not think there is anything in it worth publication, unless it may be a little account of the medical school, and you must be the judge of that.

Please present my kind regards to the Faculty.

Sincerely, your friend, J. W. FREER.

Prof. J. ADAMS ALLEN, Chicago.

P.S.—Our address is now 52 Neckar street, Stuttgart, Wurtemberg, Germany.

J. W. F.

---

HEIDELBERG, Germany, July 18th, 1867.

TO THE EDITOR OF THE CHICAGO MEDICAL JOURNAL:—

*Dear Sir:*—A graduate of Rush Medical College will always look back with pride and pleasure to the good old days passed in the pursuit of science, in the halls of his beloved *alma mater*. With such feelings, I now offer to you, for publication in your admirable journal, the report of a case which has been of considerable interest to me, hoping that, at least, it will not be unacceptable to your readers:—

N. B., *æt.* 63, entered the hospital June 24, 1867. He dates his disease back fifteen years. He had been a laboring man. Fifteen years ago, he commenced to feel occasional drawing pains, periodical in their return. From this time he began to lose flesh, and now is so emaciated as to present the appearance of a real living skeleton. He entered the hospital on account of swelling of the lower extremities and difficulty of swallowing.

About three weeks ago, he began to feel great difficulty in breathing and coughed, expectorating mucus. At about the same time, he vomited about 1½ pints of blood, followed by bile. The appetite is good and the digestion perfect, at present. This sudden hæmatemesis was probably due to ulcer of the stomach, and has but little bearing on the case. The œdema of the lower extremities appears to be owing to passive venous congestion, the veins having lost the support of the muscles, and thus, one of the most important conditions of a perfect venous circulation, namely, the contraction of the muscles, being removed. That it was due to a passive venous congestion is demonstrated by the fact of its disappearance after the patient had retained the recumbent posture for a few days.

The difficulty of breathing is caused by atrophy of the intercostals, the lungs being in a healthy condition. Heart normal; bowels constipated; urine normal. The dysphagia is extreme, the patient being totally unable to swallow solid food. The morsel remains in the œsophagus, or, rather, in the pharynx, excites a paroxysm of coughing, and is again regurgitated. Liquids still pass, but with difficulty. This dysphagia is ascribed to atrophy of the constrictors of the pharynx and œsophagus. On first inspecting the patient, one is struck with the emaciated appearance he presents. The muscles are *all* atrophied and *some* have disappeared. The arm and thigh are comparatively unaffected; the strength, in a measure, being preserved. I say comparatively, for they are not of the size and strength of those of an adult. The hands and feet, legs and forearm are greatly emaciated, and *here* the extensor muscles have suffered most. The balls of the thumbs have entirely disappeared, and no trace is to be found of the lumbricales and interossei. The fingers are bent so as to resemble claws, and the patient is unable to straighten them, or to retain them so when extended for him. The flexors are not so much affected; they outbalance the extensors, and thus the fingers are permanently flexed. The extensor communis digitorum, the abductor pollicis, and the muscles of the forearm and hand are all atrophied. The legs and feet present similar lesions. The flexors suffer here much more than in the upper extremities, the abductors being better preserved. The foot is drawn into the position of talipes varus, the patient walking on the outer border. There is no pyrexia; integument moist; pulse 72; respiration 24. The liver is normal in size, and offers no clue to any pathological change. The spleen is not enlarged, and were it not for this atrophy of the muscles, the patient would consider himself entirely well. I need not add that the face presents the same emaciated appearance as the rest of the body. The diagnosis of progressive muscular atrophy being arrived at, the following treatment was instituted:—At 8 A.M., milk; at 10 A.M., broth; at 1 P.M., egg-soup and boiled meat; at 4 P.M., coffee; at 7 P.M., egg-soup and meat. A placebo of tr. gentiani is administered four times a day.

That the prognosis is unfavorable will not surprise anybody; but that the disease should be inevitably mortal, is a conclusion in which I, for one, do not readily acquiesce. The only symptom of any gravity in the history of this case, is the hæmatemesis. This, however, seems to have been merely a symptom of some other affection, such as trauma of the pharynx or œsophagus, accidental congestion of the gastric mucous membrane, or chronic ulcer of the stomach, therefore I will leave it entirely out of the question.

That muscular tissue, after having undergone fatty degeneration, should be replaced or restored, no one will imagine. Virchow, in his able work on cellular pathology, has fully demonstrated this. To that form of fatty degeneration in which the fibrillæ themselves are softened and destroyed, he applies the term *necrobiosis.* When muscles are attacked by this form of degeneration, there is no hope of their ever again being restored to their original volume and activity, as the ultimate fibres, the fusiform cells themselves, are involved. If this were to occur suddenly, if this myomalacia were an acute process, the physician would be justified in folding his arms and doing nothing.

If the old muscle cannot be reproduced, if new muscle cannot be developed, must we allow the disease to *extend* during a period of fifteen years, to the whole muscular system? That the atrophy cannot be checked by milk and broth, egg-soup and a little tr. gentiani, is evident. What would be the result of passive exercise, electricity, beefsteaks, egg-noggs, and cod-liver oil, combined with exercise in the open air? Would a man in a state of perfect health retain his muscles in a healthy condition if kept indoors and in bed, with milk and broth for his diet? Is it reasonable to suppose that a treatment which produces atrophy in healthy muscle will check it in diseased? In these cases we have too long been accustomed to do nothing, probably on account of their rarity and chronicity. In fact, it is not long since we have understood them.

This disease was formerly described as a progressive muscular paralysis. Van Swieten was the first pathologist to correct this error. Chas. Bell and Abercrombie have reported cases. The former attributed it to nerve influence, so that when Cruveilher, in 1853, discovered that the nerves were really atrophied, he was convinced, and declared that the muscular atrophy was caused by defective innervation. Prof. Oppenheimer, of Heidelberg, shortly after, proved this theory to be utterly erroneous, the *nerve* atrophy being consequent upon the *muscular*. But Virchow gives the most lucid description. He makes a distinction between *necrobiosis*, in which the sarcous elements themselves are the seat of the disease, and *fatty degeneration proper*, in which the connective tissue is the part affected. And thus, we may at present consider ourselves acquainted with the pathology of this interesting process.

But I have already encroached too much upon your valuable space; I leave the further consideration of this subject, expecting before long to learn that some method of treatment has been devised and found adequate.

Yours, most respectfully,

SAM'L COLE, M.D.

*19 Grossemandelgasse, Heidelberg, Baden.*

